# A Novel Multifunctional β-*N*-Acetylhexosaminidase Revealed through Metagenomics of an Oil-Spilled Mangrove

**DOI:** 10.3390/bioengineering4030062

**Published:** 2017-07-09

**Authors:** Fábio Lino Soares, Joelma Marcon, Michele de Cássia Pereira e Silva, Nittaya Khakhum, Louise Teixeira Cerdeira, Júlia Ronzella Ottoni, Daniela Ferreira Domingos, Rodrigo Gouvea Taketani, Valéria Maia de Oliveira, André Oliveira de Souza Lima, João Lucio Azevedo, Jorge Luiz Mazza Rodrigues, Fernando Dini Andreote

**Affiliations:** 1 Center of Nuclear Energy in Agriculture, University of São Paulo, Piracicaba, São Paulo 13400-970, Brazil; flsoaresjunior@gmail.com; 2 Department of Biology, University of Texas, Arlington, TX 76019, USA; nittaya_kha@hotmail.com; 3 Luiz de Queiroz College of Agriculture, University of São Paulo, Piracicaba, São Paulo 13418-900, Brazil; joelma.marcon@gmail.com (J.M.); misilvafbq@gmail.com (M.C.P.S.); jlazevedo@usp.br (J.L.A.); fdandreo@gmail.com (F.D.A.); 4 Fleury Group, Jabaquara, São Paulo, 04344-070, Brazil; lcerdeira@gmail.com; 5 Center for Chemical, Biological and Agricultural Research (CPQBA), University of Campinas, Campinas, São Paulo 13148-218, Brazil; ottoni.julia@gmail.com (J.R.O.); dfdomingos@gmail.com (D.F.D.); vmaia@cpqba.unicamp.br (V.M.d.O.); 6 Embrapa Environment, Jaguariúna, São Paulo 13820-000, Brazil; rgtaketani@gmail.com; 7 Department of Biological Sciences, University of Vale do Itajai, Itajai, Santa Catarina 88302-202, Brazil; lima@univali.br; 8 Department of Land, Air and Water Resources, University of California, Davis, CA 95616, USA; 9 Environmental Genomics and Systems Biology Division, Lawrence Berkeley National Laboratory, Berkeley, CA 94720, USA

**Keywords:** bioprospection, enzyme characterization, hydrolases, microbial communities, 3D modeling

## Abstract

The use of culture-independent approaches, such as metagenomics, provides complementary access to environmental microbial diversity. Mangrove environments represent a highly complex system with plenty of opportunities for finding singular functions. In this study we performed a functional screening of fosmid libraries obtained from an oil contaminated mangrove site, with the purpose of identifying clones expressing hydrolytic activities. A novel gene coding for a β-*N*-acetylhexosaminidase with 355 amino acids and 43KDa was retrieved and characterized. The translated sequence showed only 38% similarity to a β-*N*-acetylhexosaminidase gene in the genome of Veillonella sp. CAG:933, suggesting that it might constitute a novel enzyme. The enzyme was expressed, purified, and characterized for its enzymatic activity on carboxymethyl cellulose, p-Nitrophenyl-2acetamide-2deoxy-β-d-glucopyranoside, p-Nitrophenyl-2acetamide-2deoxy-β-d-galactopyranoside, and 4-Nitrophenyl β-d-glucopyranoside, presenting β-*N*-acetylglucosaminidase, β-glucosidase, and β-1,4-endoglucanase activities. The enzyme showed optimum activity at 30 °C and pH 5.5. The characterization of the putative novel β-*N*-acetylglucosaminidase enzyme reflects similarities to characteristics of the environment explored, which differs from milder conditions environments. This work exemplifies the application of cultivation-independent molecular techniques to the mangrove microbiome for obtaining a novel biotechnological product.

## 1. Introduction

Mangroves are highly productive coastal ecosystems located on transition zones between terrestrial, freshwater and marine environments. These ecosystems are characterized by periodic tidal flooding, leading to highly variable environmental conditions, such as salinity and nutrient availability [1]. The mangrove studied here is especially unique as it is highly contaminated owing to an oil spill of 35 million gallons in 1983, which resulted in extensive damages to this ecosystem [2]. The decomposition of plant (leaves, stumps, roots) or animal (shells of crab, shrimp, clams) residues under these conditions are very slow, acting as a selective pressure on the evolution of enzymes involved in mineralization processes [3,4,5]. Although the composition of microbial communities residing in different mangroves has been depicted in other studies [6,7,8], the functional profiles of microbial communities in this ecosystem are yet to be properly described.

In mangroves, a high abundance of hydrolases has been reported in bacterial isolates [9]. Hydrolases have a key role on organic matter cycling. Among these, enzymes with β-N-Acetylhexosaminidase activity (EC 3.2.1.52) are distributed across three different families of glycoside hydrolases, GH3, GH20 and GH84 [10] (CAZy database-http//: www.cazy.org) [11]. Glycoside hydrolases are a group of enzymes that promote the hydrolysis of the bond between two or more carbohydrates, or between a carbohydrate and another molecule. The glycoside hydrolase family 3 includes β-*N*-acetylhexosaminidases and β-glucosidases that are mainly produced by bacterial cells, although there are a few descriptions of those detected in eukaryotes [12]. Enzymes belonging to this family behave as typical exoenzymes, catalysing the cleavage of terminal β-1,4 non-reducing ends of biopolymers [13,14]. These important glycoside hydrolases are known to occur upon two of the most abundant sources of carbon, cellulose and chitin [15], both present in high amount in the organically enriched soils from mangroves.

The use of culture-independent approaches, such as metagenomics, provided complementary access to the vast majority of the microbial diversity in the environment [2,16]. The generation of metagenomic libraries, in combination with the possibility to sequence large inserts from fosmid clones, constitutes a successful initial bioprospection approach. It allows the description and characterization of new enzymes and supports robust inferences on the taxonomical affiliation of organisms harboring important features in the environment [17,18]. Given the great importance of microorganisms in enzyme production, it is clear the interest in the application of methods to explore their biotechnological potential [19,20], allowing the access to the wealth of microbial resources contained in the metagenome of mangroves. Thus, in this study we performed a functional screening of fosmid libraries obtained from an oil contaminated mangrove site aiming to identify clones expressing hydrolytic activities, followed by subcloning and sequencing of the active insert. We succeeded in the retrival of a novel enzyme of the GH3 family, classified as a β-*N*-acetylhexosaminidase. The enzyme was heterologous expressed and characterized.

## 2. Materials and Methods

### 2.1. Metagenomic Fosmid Library Construction

The study site is a mangrove located in the city of Bertioga, state of São Paulo (23°53′49′′ S, 46°12′28′′ W). The mangrove has been extensively characterized regarding the effects of petroleum contamination on its microbial community [2,6,21,22]. Soil samples were collected in triplicate, at 30 cm deep using sterile polypropylene tubes, limiting the air entrance to a minimum. These samples were used for DNA extraction and fosmid library construction as described in Vasconcellos et al. [23], using the Ready-Cloning pCC2FOS Copy Control kit (Epicentre, Madison, WI, USA), following manufacturer’s recommendations. A total of 12,960 clones were obtained, with insert size ranging between 30 and 40 kb, and 30 clones were randomly selected for library validation based on insert restriction analysis.

### 2.2. Screening for Cellulase Activity

*Escherichia coli* transformants were replicated on minimal salt medium (NaNO_3_ 6.0 g·L^−1^, KH_2_PO_4_ 1.5 g·L^−1^, KCl 0.5 g·L^−1^, MgSO_4_ 0.5 g·L^−1^, FeSO_4_ 0.01 g·L^−1^ and ZnSO_4_ 0.01 g·L^−1^) amended with 1% carboxymethyl-cellulose (CMC) as sole carbon source and incubated at 37 °C for 72 h [4]. Plates were stained with an aqueous iodine solution (1%) and clones showing degradation halos were selected as putatively positive hits [24], as halo formation around the colonies indicate degradation of substrates due to hydrolytic activity. Clones were confirmed as positives through two additional consecutive cultivations at the same growth rate and testing conditions.

### 2.3. Fosmid DNA Extraction and Sequencing

Fosmid DNA was extracted using the Qiagen Large-Construct Kit (Qiagen Inc., Hilden, Germany), following the manufacturer’s recommendations. DNA was quantified using the Quant kit ITTM-dsDNA-BR assay (Invitrogen, São Paulo, Brazil) and a total of 1.0 μg was used for sequencing with the IonTorrent PGM platform (Life Technologies, Camarillo, USA). DNA library construction and enrichment were performed with the ION Xpress DNA Fragment Library and ION XpressTM Template v2.0 kits (Life Technologies, Camarillo, USA), respectively. Samples were linked to beads using the ION touch device (Life Technologies, Camarillo, USA) and injected into the 316-chip for sequencing.

### 2.4. Sequence Assembly and Analysis

Readings shorter than 100 bp or presenting low quality bases (Phred score < 20) were removed from subsequent analyses. The remaining sequences were mapped against the pCC2FOS vector sequence and genomic DNA from the host strain *E. coli* EPI-300 and excluded from downstream analyses. Assembly and mapping of high quality reads were performed in the CLCbio Genomics Workbench (Qiagen Inc., Hilden, Germany) using the de novo assembly tool with the following the parameters: (i) Cost Mismatch: 2; (ii) Interaction Cost: 3; (iii) Deletion Cost: 3; (iiii) Fraction Length: 0.3; (iiiii) Similarity Fraction: 0.6. The final contig, named 131-H9, was submitted to the Rapid Annotation Subsystem Technology-server (http://rast.nmpdr.org) [25], functionally annotated using the tool Microbial Finding Gene System-glimmer, and the annotated sequences were further manually checked. Open Reading Frames (ORFs) were analyzed by blastx [26] against GenBank (nr/nt), Protein Data Bank and SwissProt databases. The sequence is available at the Genbank under the accession number KX599540.

### 2.5. Inferences on the Structure of the Enzyme

In order to evaluate the potential novelty of the enzyme structure, the primary protein sequence was compared using the BlastP algorithm [27] to the non-redundant Genbank protein database (National Center for Biotechnology Information—NCBI). The thirty most similar enzyme sequences in the database were recovered and used for a phylogenetic assessment, using the Grishin distance [28], an estimation of divergence and Fast Tree Minimum Evolution [29]. Additionally, family domains and their functions were searched using CD-Search at NCBI [30] and CDART—domains architecture [31]. Sequences coding for secretory signals were evaluated by SignalP 4.1 [32]. In order to determine the enzyme structure and potential active sites, tridimensional models were predicted using the Swiss-Model [33] and Phyre [34] and visualized with the Swiss-PdbViewer v4.1.0 [35]. To identify the nucleophile residue and the catalytic dyad in the protein, the primary sequence was surveyed for the consensus motif and the 3D structure was predicted. The Phyre algorithm considered *Bacillus subtilis N*-acetylglucosaminidase—BsNagZ as template (c3bmxB, resolution 1.40 Å) for modeling the Nag_Mgrv-Met protein, while the Swiss-Model used the mutant (N318D) version (3lk6.1.A) of the same gene.

### 2.6. Sub-Cloning of the β-N-Acetylhexosaminidase-Related ORF

To identify the gene coding for the β-*N*-acetylhexosaminidase, a primer pair was designed (Sigma-Aldrich, St. Louis, MO, USA): BeHexo_F_express: 5′TATAAAAAGCTTGGATGACCTTGCCCCGAAGG3′ (containing the site for HindIII cleveage) and the BeHexo_R_express: 5′ATAATACTCGAGTCAGGATTGGGGCGGATTC3′ (containing the site for XhoI cleveage). PCR reaction was carried out by an initial denaturation step at 94 °C for 3 min, followed by 35 cycles of 94 °C for 60 s, 60 °C for 30 s and 72 °C for 30 s. The PCR product was purified with the QiAquick PCR Purification kit (Qiagen Inc., Germantown,, USA) and the expected fragment (1065 bp) was determined by electrophoresis of the amplified PCR product using 1% agarose gel in 1× Tris-Acetate-EDTA buffer, stained with ethidium bromide (1%), and visualized under UV light. The amplified PCR product and the expression vector pET28a were cleaved with the same endonucleases, HindIII and XhoI (New England Biolabs, Ipswich, MA, USA), and further ligated in a 10 μL-volume reaction containing 1× T4 DNA ligase buffer, 400 U/μL of T4 DNA ligase (New England Biolabs, Ipswich, MA, USA), 40 ng of linearized pET-28a plasmid and 100 ng of PCR product, following incubation for 12 h at 16 °C. Circularized plasmids were then inserted into the *E. coli* strain BL21 DE3 by electroporation (1.8 KV, 1.800 Ω). Transformant colonies grown on Luria Bertani medium with kanamycin (50 μg/mL) were selected for plasmid DNA extraction using QiAprep Spin Miniprep kit (Qiagen Inc., Germantown, USA). Screening for the presence of insert was done by PCR with the same primers as mentioned previously. Selected inserts were sequenced using the Sanger method at the Genomics Core Facility, University of Texas at Arlington, to confirm the identity of the cloned sequence.

### 2.7. Purification of the 6xHis-Tagged β-N-Acetylhexosaminidase

*Escherichia coli* cells hosting the cloned sequence were cultivated in LB medium supplemented with kanamycin (50 μg/mL) at 37 °C overnight. Culture was diluted (1:10) to an optical density of (600 nm) using the same medium with addition of 50 μL of IPTG (1 mM) as inducer. Cells were cultivated for 2 h at 37 °C and harvested by centrifugation (4500× *g* for 30 min at 4 °C). The pellet was suspended in 10 mL of lysis buffer (100 mM NaH2PO4; 10 mM Tris-HCl; 8 mM urea; pH 8.0 adjusted with NaOH), supplemented with 1 mg/mL of lysozyme. The mixture was incubated overnight at 4 °C, and centrifuged (10,000× *g* for 30 min at 4 °C). The clear lysate was filtered through a 0.45 μm cellulose membrane, amended with 1 mL of Ni-nitrilotriacetic acid agarose slurry (NI-NTA, Qiagen Inc., Germantown, USA) and incubated for 30 min at 4 °C.

The β-*N*-acetylhexosaminidase was purified by gravitational flow purification using a 15 mL-column for collecting the flow through (Qiagen Inc., Germantown, MD, USA). Fractions were collected under different pHs using a denaturing condition buffer (100 mM NaH_2_PO_4_; 10 mM Tris-HCl; 8 M Urea), as follows: 1× lysis buffer (pH 8.0), 2× wash buffer (pH 6.3); 4× elution buffer (pH 5.9); 4× elution buffer (pH 4.5). Eluted proteins were transferred to buffer native condition (50 mM NaH_2_PO_4_; 300 mM NaCl; 250 mM Imidazole; pH 8.0) and concentrated using the Amicon ultra-0.5 centrifugal filter with 30 K cut-off value (Millipore, Billerica, MA, USA). All fractions were mixed with 5× sample buffer (250 mM Tris-HCl pH 6.8, 500 mM DTT, 10% SDS, 0.1% bromophenol blue, 50% glycerol), heated to 95 °C for 10 min, and electrophoresed through 8–12% SDS-PAGE gel (Nusep, Bogart, GA, USA) in 1× Tris-glycine electrophoresis running buffer (3.02 g Tris base, 14.4 g glycine, 1 g SDS in 1 L dH2O). The gel was stained overnight in 15 mL of coomassie brilliant blue NuBlu Express stain (NuSep Ltd., Homebush, Australia). The protein marker was the PageRuler broad range unstained protein ladder 10–200 kDa (Thermo Scientific, Waltham, MA, USA). The gel was visualized under white light with the GelLogic 212 PRO Carestream (Carestream Health, New Haven, CT, USA). Protein concentrations were determined with protein quantification by Pierce BCA Protein Assay Kit (Thermo Scientific, Pierce, Rockford, IL, USA) with bovine serum albumin as the standard.

### 2.8. Assays for Characterization of the Enzymatic Activity

The activity of the purified β-*N*-acetylhexosaminidase was determined at different temperatures (10 to 60 °C), pH values (2.0 to 9.0) and salt concentrations (0.5 and 2.0 M NaCl), from 0 to 24 h. These activities were estimated by the release of p-nitrophenol through the hydrolysis of p-Nitrophenyl-2acetamide-2deoxy-β-d-glucopyranoside (pNP-GlcNac), p-Nitrophenyl-2acetamide-2deoxy-β-d-galactopyranoside (pNP-GalNac), and 4-Nitrophenyl β-d-glucopyranoside (pNP-Glc) (Sigma-Aldrich, St. Louis, MO, USA). These substrates are oligosaccharides with specific variations in their carbon chains according to the respective enzyme activity. Results were expressed as the percentage of residual activity as nmol/min/ml. All assays were conducted in triplicate, in 100 μL-reaction mixture containing 0.5 mM·L^−1^ pNP-GlcNac, pNp-GalNac or pNP-Glc, incubated under distinct conditions. Reactions were carried out for 30 min, and stopped with the addition of 900 μL Sodium Borate buffer 0.2 M (pH 10.5) [34]. The amount of p-nitrofenol (pNP) released was estimated by the absorbance of the final solution at λ = 405 nm. One unit (U) of β-*N*-acetylhexosaminidase activity was defined as the amount of enzyme that produced 1 μmol·L^−1^ pNP released per minute. The determination of the optimum temperature for the enzymatic activity was conducted using Tris-HCl buffer, pH 6.8. Under the optimal determined temperature, the enzymatic activity was assayed under different pH values (2–9) using 50 mM·L^−1^ Glycine-HCl buffer (pH 2–4), 50 mM·L^−1^ sodium acetate buffer (pH 4–6), 50 mM·L^−1^ sodium phosphate buffer (pH 6–8) and 50 mM·L^−1^ Tris-HCl buffer (pH 8–9). Finally, under the optimal temperature and pH, the effects of salinity (0.5–2 M of NaCl) on the enzyme activity were evaluated. To examine the stability of the enzyme, it was incubated under optimal conditions—as previously determined (30 °C, pH 5.0 and 0.5 M of NaCl)—and the residual activity was determined every 30 min during 24 h.

## 3. Results

### 3.1. Detection and Sequencing of the Active Clone

The hydrolytic activity on CMC, through formation of a degradation halo around colonies of bacterial transformants, allowed identification of a positive clone named 131-H9 (Figure S1). The sequencing approach resulted in the generation of 1,175,586 reads with an average size of 198 bp. After quality control (removal of low quality sequences and those from vector and *E. coli*), a total of 786,468 sequences were bined into a single contig of 39,586 bp in length (Figure S2). Annotation based on similarities with the Protein Data Bank (PDB) and the SwissProt databases in the RAST platform identified 18 ORFs (Open reading frames; each >1000 bp). Among them, one stood out as it was related to a β-*N*-acetyl-hexosaminidase-coding gene. This ORF, named Nag_Mgrv-Met (EC3.2.1.52), had 1065 nucleotides in length with average GC content of 58.5%. Its translated sequenced corresponded to 355 amino acids with homology to members of the super family GH3.

### 3.2. Prediction of the Enzyme Structure

**Figure 1:**
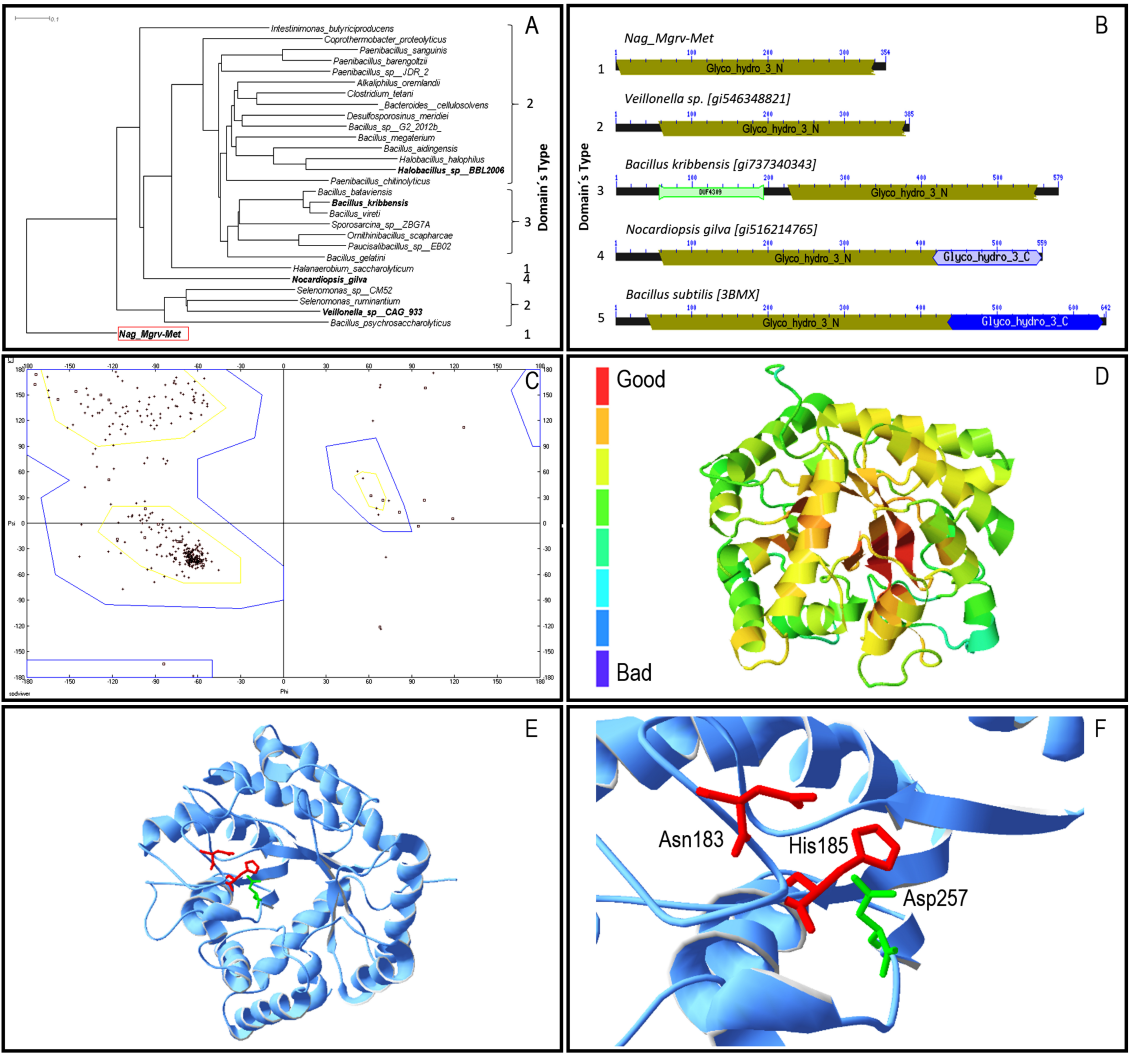
Molecular characterization of protein Nag_Mgrv-Met Panels: (**a**) Phylogenetic tree of Nag_Mgrv-Met and homologues was built using Grishin distance [28] and Fast Tree Minimum Evolution [29]. Scale bar represents number of changes per aminoacid position; (**b**) Domain architecture—domain modular organization predicted by CDART [31]. Numbers indicate proteins; bars in dark grey: relative position and size of GH3_N; light/dark blue: GH3; light green: C terminal and unknown function (DUF4309); (**c**) Ramachandran plot–dyad chain Phi e Psi determined for Nag_Mgrv-Met, predicted by the Phyre’s model, which considered Bacillus subtilis *N*-acetylglucosaminidase-BsNagZ [35] as template (c3bmxB); (**d**) ProQ2 [36]: quality evaluation of Nag_Mgrv-Met model predicted by Phyre’s model; (**e**,**f**) Nag_Mgrv-Met–Phyre’s models visualized in Swiss-Model and Protein Data Bank (PDB) viewer v410 [37]. In red the Asparagine 183 (Asn) e Histidine 185 (His) residues, and in green the Aspartate 257 (Asp) residue.

Primary sequence analysis through BlastP revealed that Nag_Mgrv-Met has low identity to proteins deposited at non-redundant Genbank/NCBI database (66 million sequences—April 2015). For instance, the highest Max Score was assigned to a glycosyl hydrolase from Veillonella sp. CAG:933 (GI:546348821), with only 38% of identity among the 97% residues compared. The distinct aminoacid composition of Nag_Mgrv-Met was also evident at phylogenetic level (Figure 1a), where it was placed as outgroup among its most similar sequences.

In total, four distinct domains were identified among the sequences analysed (Figure 1b), which were grouped in five architecture types. Nag_Mgrv_Met was placed in architecture type 1 group, which contains the family 3-N domain of glycosyl hydrolases (GH3_N). The catalytic (β/α)8 barrel structure found in GH3_N (N-terminal domain) contains a conserved aspartate nucleophile and a catalytic histidine/aspartate dyad on the flexible loop which can be identified by the consensus motif [KH(F/I)PG(H/L)GXXXXD(S/T)H] (histidine/aspartate dyad highlighted in underline). Modeled structures were quite identical, with only 1.78 Å spatial distance among 336 α-carbons considered by the Swiss-PdbViewer. BsNagZ is a multiple domain protein (Figure 1b), thus only part of it (GH3_N domain) was considered for modeling, which was limited to 30–32% (depending on the algorithm applied) identical to the Nag_Mgrv-Met protein. Despite the low identity, the model´s quality was suitable for further analysis with a determined GMQE (Global quality estimation score) of 0.69 as calculated by Swiss-Model (values can vary between 0 and 1). Furthermore, a Ramachandran plot analysis using the Phyre 3D predicted model indicated that 92.3% of the residues are found to be in favored region, with only 2.4% in outlier region (Figure 1c). ProQ2 results were similar to GMQE, placing the predicted Nag_Mgrv-Met 3D between moderate to good quality (Figure 1d).

Catalytic sites were confirmed on the Phyre predicted model (Figure 1e,f). The consensus motif [KH(F/I)PG(H/L)GXXXXD(S/T)H] containing the dyad Aspartate/Histidine (underlined residues), which is conserved among β-*N*-acetylglucosaminidases of family 3 glycosidases, was identified in the Nag_Mgrv-Met protein, although the sequence found [172-KHFPGHGSALGNTH-185] (underlined Asparagine/Histidine dyad ) presented a substitution of the negatively charged Aspartate residue by the non-charged polar sidechain Asparagine residue.

### 3.3. Purification and Characterization of the β-N-Acetylhexosaminidase

**Figure 2:**
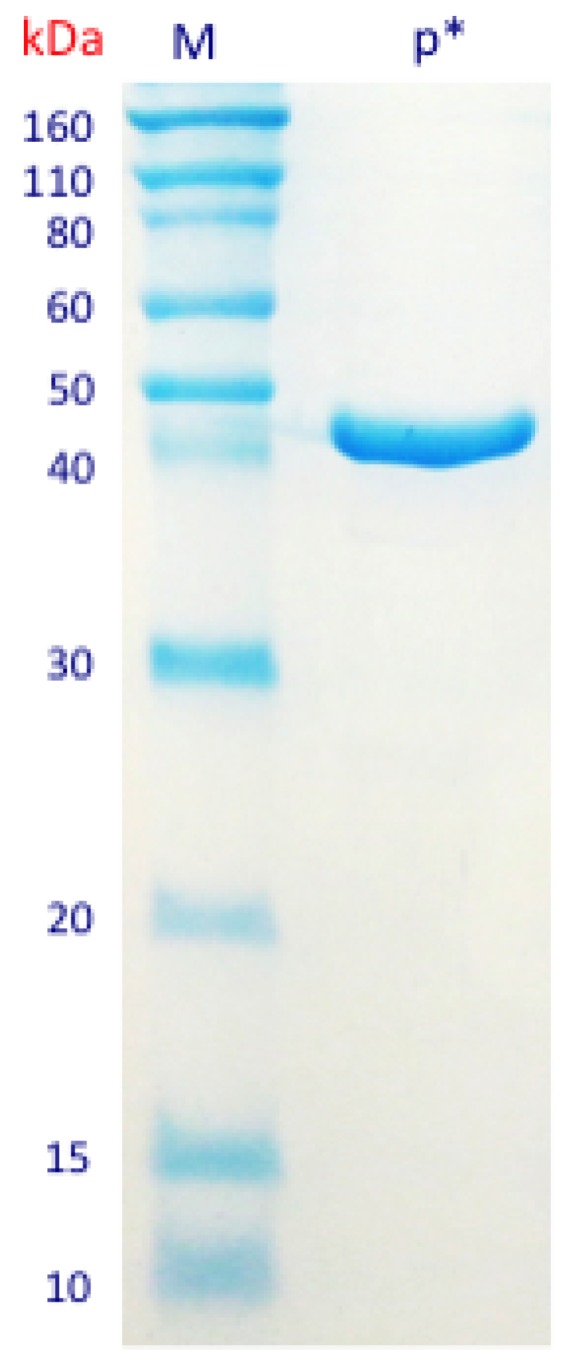
SDS-PAGE of the isolated purified protein in kDa M: Molecular marker; p* Purified protein band.

The first step in the enzyme purification process was conducted by cloning the sequence corresponding to β-*N*-acetylhexosaminidase (1065 bp) into the plasmid vector pET28a and transforming *E. coli* BL21 host cells. The cloned DNA was sequenced to confirm sequence identity and insertion of the histidine-tag (6xHis-tag) in the end of the target sequence. The expression of the cloned sequence was induced and the sequence encoding for 6x histidine-tag (6xHis-tag)—linked to the targeted sequence—was used to facilitate the protein purification by binding to Ni-NTA resins. The candidate β-*N*-acetylhexosaminidase was purified from the total protein extract in a process monitored by quantification and visualization of the protein patterns (Figure S3). The final product was obtained and a single band was observed in the SDS-PAGE, with deduced molecular mass of 43 kDa—corresponding to the estimated size of the protein (Figure 2).

The hydrolytic activity of the enzyme was observed upon three substrates: GlcNac, GalNac and pNP-Glc. Average absorbance values were obtained for all of them (Figure S4), supporting the indication for a novel hydrolytic scaffold. The highest activity was observed for pNP-GlcNac (74 mmol/min/mL), followed by pNP-GalNac (60 mmol/min/mL) and pNP-Glc (15 mmol/min/mL) (Table 1).

**Figure 3:**
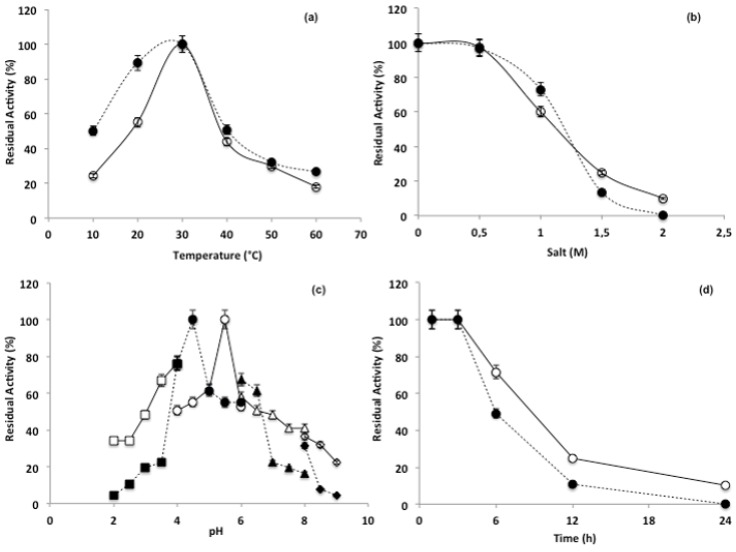
Characterization of optimum enzymatic conditions calculated as percentage of residual activity: (**a**) In relation to temperature (10 to 60 °C); (**b**) in relation to sodium chloride concentrations (0 to 2 M); (**c**) in relation to pH (20 to 100); (**d**) in relation to stability (0 to 24 h) Continuous line represents β-*N*-acetylhexosaminidase activity in relation to pNP-GlNac; Dotted line represents β-*N*-acetylhexosaminidase activity in relation to pNP-GalNac.

The putative new β-*N*-acetylhexosaminidase presented 98% of its potential activity when incubated at 30 °C, with a marked decreased activity for substrate pNP-Glc (13.8%), followed by substrates pNP-GlcNac (46.3%) and pNP-GalNac (50.8%), in temperatures lower than 20 °C and higher than 40 °C (Figure 3a). Salinity did not preclude the enzymatic activity when NaCl concentrations were lower than 0.5 M, and all substrates demonstrated residual activity varying from 100 and 97.5% (GlcNac), 97% (GalNac) and 93.1% (Glc) (Figure 3b). However, with increments from 1 to 2 M the activity decresead, reaching average values of 50% (1 M) and 25% (1.5 M). A similar approach revealed distinct peaks for activity along the pH gradient tested. Optimal activites were observed at pH 5.5 for GlcNac, pH 4.5 for GalNac and pH 5.0 for pNPGlc (Figure 3c). The stability test indicated that the enzyme activity is impaired after 3 h of incubation, after which the activity decreased linearly along the incubation period (Figure 3d). After an incubation period of 6 h, the percent activity was decreased to 71.6% for GlcNac, 49.2% for GalNac and 11.5% for Glc.

## 4. Discussion

Mangroves are characterized by high rates of nutrient turnover and organic matter recycling between the ocean and terrestrial habitats [38,39,40]. Remarkably, the mangrove sampled in this study is unique as it is highly contaminated owing to an oil spill of 35 million gallons in 1983. Such an event resulted in extensive damage to this ecosystem and today still, its microbiome is far from recovered [2].

The microbiome of extreme environments such as mangroves is exceedingly diverse, allowing the performance of unique functions. Multiple studies have shown the importance of mangroves as source of important enzymes, which may help in the mineralization of organic matter, modulating nutrient cycling in soils [41,42,43]. Previously, we have isolated and described the bacterium *Bacillus thuringiensis* BrMgv02-JM63 from the same oil-contaminated sediment examined in this study and its sequenced genome revealed genes associated with chitinolytic activity [15]. Furthermore, Dinesh et al. [44] isolated and sequenced the genome of a bacterium affiliated to the genus *Mangrovimonas*, from an estuarine mangrove, and found genes involved in degradation of xylan. Considering the above findings, further studies on the degradation of organic matter and identification of novel endo- and exoglicolytic enzymatic activities are of great importance to environmental sciences and industry, respectively.

Our metagenomic approach allowed us to obtain a fosmid clone displaying hydrolytic activity towards the substrated carboxymethyl cellulose, named here Nag_Mgrv-Met gene, which codifies a β-*N*-acetyl-hexosaminidase enzyme (EC 3.2.1.52) that belongs to Type I (GH3_N). Housing the active site pocket and the catalytic nucleophile, GH3_N was first described as (alpha/beta)8 TIM-barrel domain on the multiple domain barley beta-d-glucan exohydrolase (ExoI) [45]. Moreover, the low identity observed allowed recognition of Nag_Mgrv-Met as a novel protein. This characteristic is one of the criteria considered to assign novelty to metagenomic hydrolases, as previously reported to a β-glucosidase from soil [46] a glycosidase from rumen [47], a marine esterase [48] and a xylanase/cellulase derived from farm compost [49], which showed 74, 68, 27, 25% sequence identity, respectively. Aside from the low identity to previously sequenced GH3’s, the activity on pNP-GalNac also makes this enzyme unique.

GH3 glycoside hydrolase family is broadly distributed in bacteria, fungi and plants. Studies involving isolation and purification of β-glucosidases from microorganisms date from the 1990s [50]. It includes exo-acting β-d-glucosidases, α-l-arabinofuranosidases, β-d-xylopyranosidases and *N*-acetyl-β-d-glucosaminidases [51]. Thus, these enzymes have a wide range of cellular functions, such as plant and bacterial cell wall remodeling, energy metabolism, pathogen defense and biomass degradation. Some of GH3 enzymes are characterized as bifunctional, for example, Nag3 from *Cellulomonas fimi* was described with *N*-acetyl-β-d-glucosaminidase and β-glucosidase activities [12]. The majority of GH3 with *N*-acetyl-β-d-glucosaminidase activity from Gram-negative bacteria are single-domain enzymes, as verified for Nag_Mgrv-Met. Today, more than seventy-four thousand proteins at GenBank/NCBI contain GH3_N (85% bacterial), which are organized in 145 distinguished domain architectures [31]. Single GH3_N domain proteins, as Nag_Mgrv-Met, are the second most abundant ones (12,228 non-redundant sequences). Nevertheless, our target primary sequence is quite rare and not fully described.

Our newly discovered enzyme Nag_Mgrv-Met was able to hydrolyze pNP-GlcNac, pNP-Glc and CMC substrates, thus presenting β-*N*-acetylglucosaminidase, β-glucosidase and β-1,4-endoglucanase activities. A substitution mutation leading to Asn rather than Asp could be responsible for these activities. Supporting these findings, an analysis of *N*-acetyl-β-d-glucosaminidases from *Bacilus subtillus* [52] and the Gram-negative bacteria *Vibrio furnisii* [53,54] and *Salmonella typhimurium* [35], indicated that although the catalytic nucleophile of glycosyl hydrolase family 3 enzymes is well conserved, the identity and location of the general acid/base residue is not. Moreover, detailed dynamic mechanism of substrate binding indicated that histidine undergoes significant structural changes during catalysis, which provides support for the proposal of a noncanonical histidine acting as the general catalytic acid/base residue within NagZ and its orthologs [35].

Corroborating the results obtained from the characterization of the novel enzyme and its optimal activity (30 °C; pH 5.0; 0.5 M; 3h/stability), Ogawa et al. [55] described similar temperature and pH optimal conditions (37 °C and pH 5.5). The pH values ranging from 4.0 to 6.0 are known to be typical for the substrates tested here [56,57]. The optimal temperature for enzymatic activity is modulated according to the environmental conditions [58,59]. Considering that the average temperature in the sampled mangrove ranges from 25 to 30 °C [4], it was expected that the enzyme activity would peak at these temperatures. Similar results were observed regarding salinity, allowing us to demonstrate that the described enzyme still retained 100% of activity at 0.5 M of salt (NaCl) (although decreasing afterwards), correlating to mangrove conditions. The results of the stability test indicates that the enzyme is highly stable over time and departs from the general assumption that frequent changes in environmental conditions in marine systems result in decreases in enzyme activity [60]. In summary, the characterization of a novel β-*N*-acetylglucosaminidase enzyme reflects similarities to the environment explored, which differs from environments under the influence of milder conditions, demonstrating the importance of the present study and how we can combine classical and molecular techniques to explore new microbial enzyme from the mangrove microbiome.

## Figures and Tables

**Table 1:** Activity variation of the distinct substrates tested. The activity is reported as mmol/min/ml in relation to the final percentage. (pNP-GlcNac: 4-nitrophenyl *N*-acetyl-B-d-glucosaminide; pNP-GalNac: 4-nitrophenyl *N*-acetil-B-d-galactosaminide and pNP-Glc: 4-Nitrophenyl-β-d-Glucoside).

Carbon Source	Activity (mmol/min/mL)	Relative Activity (%)
pNP-GlcNac	74	100
pNP-GalNac	60	81
pNP-Glc	15	20

## Supplementary Materials

The following are available online at www.mdpi.com/2306-5354/4/3/62/s1, Figure S1: Cultivation plate containing the clone able to degrade carboxymethyl-cellulose (CMC); Figure S2: Annotation of open reading frames (ORF) belonging to the largest mapped contig (22,143 bp) Arrows in colors show the orientation (right/left) indicating the reading position of each ORF The β-*N*-acetyl-hexosaminidase enzyme is shown in red, with 1065 bp and match with BglX (β-glicosidases) and affiliated with family GH3 of glycoside hydrolases Numbers 1 to 1065 represent the size of the fragment in base pairs; Figure S3: SDS-PAGE after elutions of the purified protein, in kDa M: Molecular marker; pET28a: Plasmidial vector; pellet: Pellet with fase separation; sup: supernatant after centrifugation (fases separation); flow: lise buffer (pH 8.0); c1 and c2: washing buffer (pH 6.3); d1 and d4: elution buffer 1 (pH 5.9); e1 and e4: elution buffer 2 (pH 4.5); Figure S4: Absorbance values obtained for the distinct substrates tested Bars represent the maximum absorbance values reached for each substrate: pNP-Glcnac (0567), pNP-GalNac (0458) and pNP-Glc (0137).
