# Supplementary figures and images for: A Novel Multifunctional β-N-Acetylhexosaminidase Revealed through Metagenomics of an Oil-Spilled Mangrove
Source: Bioengineering (Basel). 2017 Jul 9;4(3):62. doi: 10.3390/bioengineering4030062 (PMC5615308; doi:10.3390/bioengineering4030062)

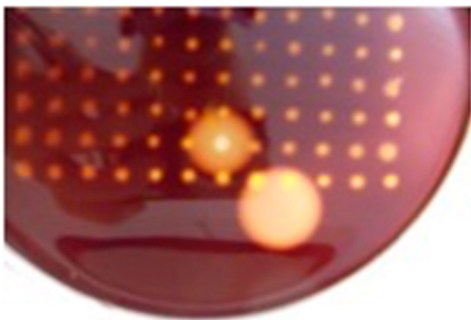

Figure S1.

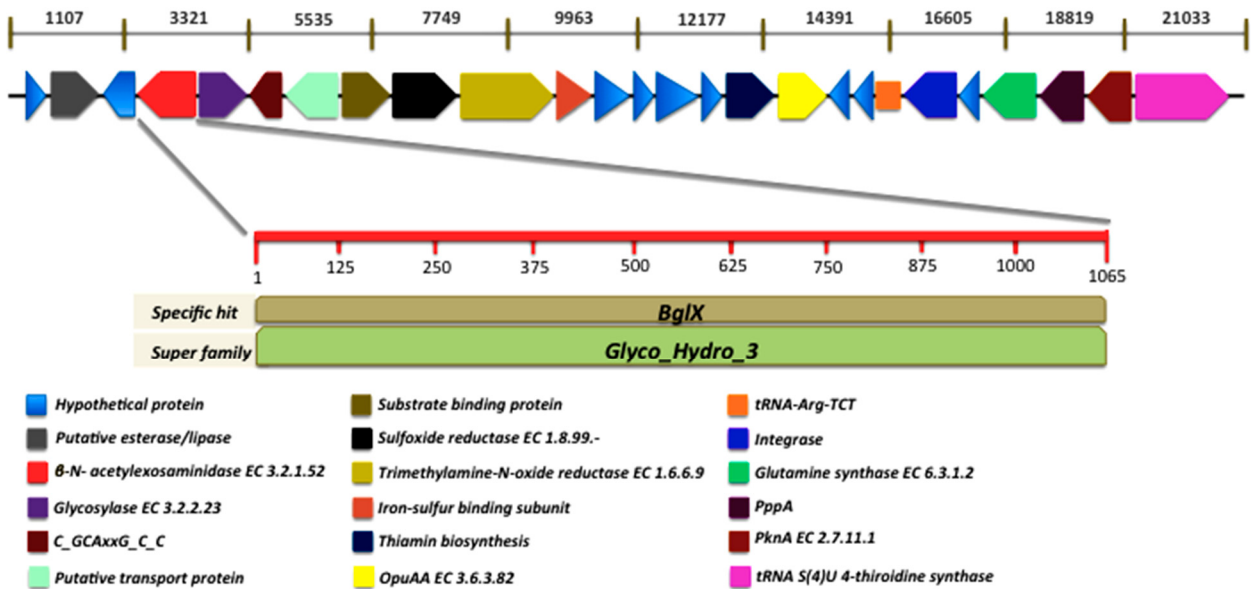

Figure S2.

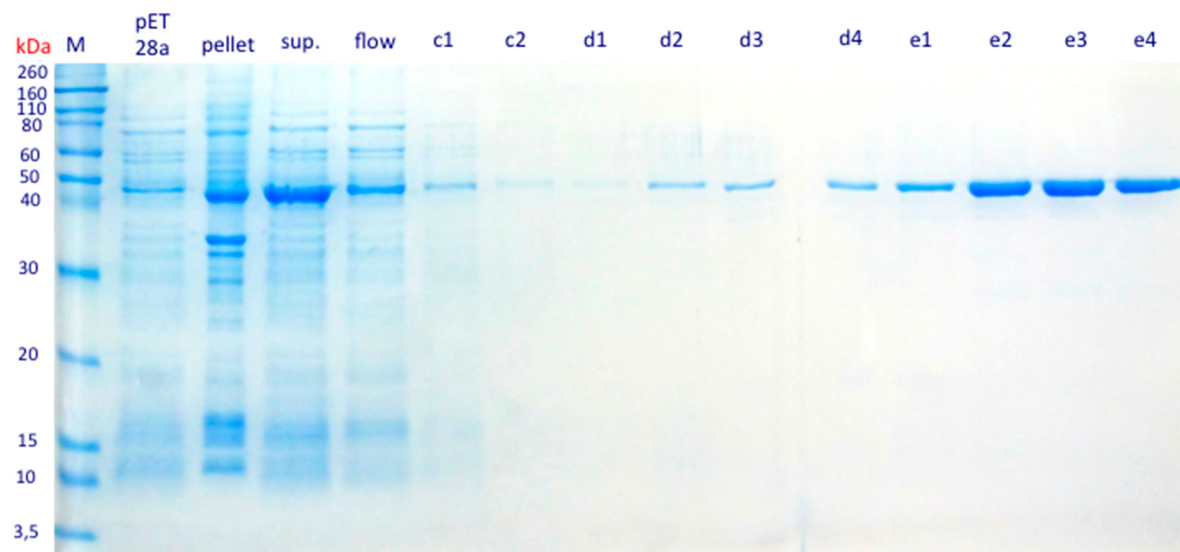

Figure S3.

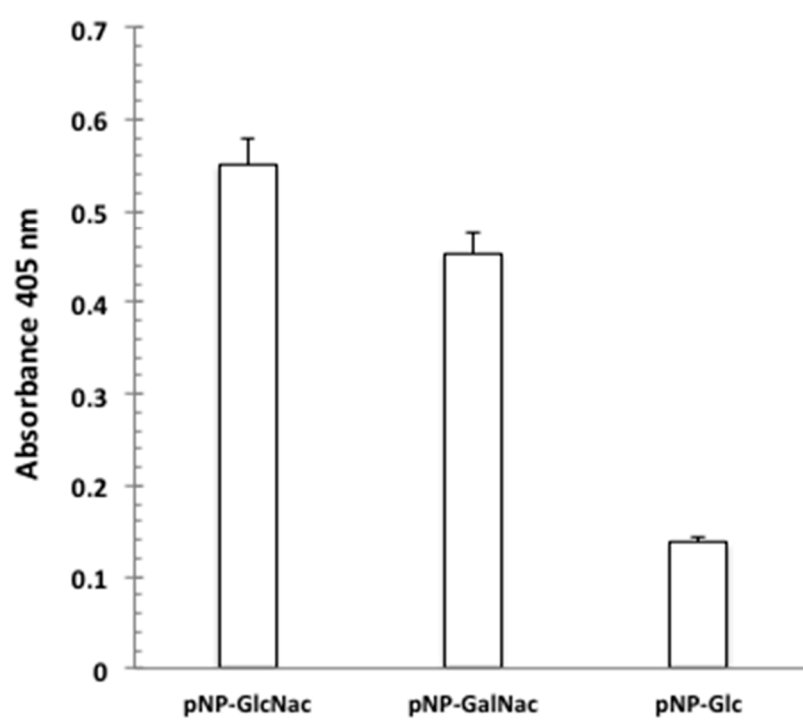

Figure S4.

Supplement: Supplementary file 1 [file bioengineering-04-00062-s001.pdf]
